# Advances of MR imaging in glioma: what the neurosurgeon needs to know

**DOI:** 10.1007/s00701-025-06593-6

**Published:** 2025-06-21

**Authors:** Anna Falk Delgado

**Affiliations:** Department of Clinical Neuroscience, Karolinska Institutet, Stockholm, Sweden

**Keywords:** 5ALA - 5-aminolevulinic acid, CBV-cerebral blood volume, CEST-chemical exchange saturation transfer, MRI-magnetic resonance imaging, DTI-diffusion tensor imaging, FMRI-functional MRI, GKS-gamma knife surgery, LITT-Laser induced thermal therapy, T2FLAIR-T2 fluid attenuated inversion recovery, RT-radiation therapy

## Abstract

Glial tumors and especially glioblastoma present a major challenge in neuro-oncology due to their infiltrative growth, resistance to therapy, and poor overall survival—despite aggressive treatments such as maximal safe resection and chemoradiotherapy. These tumors typically manifest through neurological symptoms such as seizures, headaches, and signs of increased intracranial pressure, prompting urgent neuroimaging. At initial diagnosis, MRI plays a central role in differentiating true neoplasms from tumor mimics, including inflammatory or infectious conditions. Advanced techniques such as perfusion-weighted imaging (PWI) and diffusion-weighted imaging (DWI) enhance diagnostic specificity and may prevent unnecessary surgical intervention. In the preoperative phase, MRI contributes to surgical planning through the use of functional MRI (fMRI) and diffusion tensor imaging (DTI), enabling localization of eloquent cortex and white matter tracts. These modalities support safer resections by informing trajectory planning and risk assessment. Emerging MR techniques, including magnetic resonance spectroscopy, amide proton transfer imaging, and 2HG quantification, offer further potential in delineating tumor infiltration beyond contrast-enhancing margins. Postoperatively, MRI is important for evaluating residual tumor, detecting surgical complications, and guiding radiotherapy planning. During treatment surveillance, MRI assists in distinguishing true progression from pseudoprogression or radiation necrosis, thereby guiding decisions on additional surgery, changes in systemic therapy, or inclusion into clinical trials. The continued evolution of MRI hardware, software, and image analysis—particularly with the integration of machine learning—will be critical for supporting precision neurosurgical oncology. This review highlights how advanced MRI techniques can inform clinical decision-making at each stage of care in patients with high-grade gliomas.

## Introduction

In the context of glial tumors, high-grade gliomas (HGGs) are among the most aggressive and lethal primary brain tumors, posing significant diagnostic, therapeutic, and prognostic challenges. Readily visible on standard magnetic resonance imaging (MRI), these tumors nonetheless remain difficult to treat due to their infiltrative nature, molecular heterogeneity, and poor response to therapy. Despite decades of research and the development of multimodal treatment regimens, including maximal safe surgical resection, concurrent chemoradiotherapy, and maintenance chemotherapy, patient outcomes remain dismal, with median survival for glioblastoma still under two years in most series [35, 42].

As of the 2021 World Health Organization (WHO) Classification of Central Nervous System Tumors, high-grade gliomas are no longer defined by histology alone, but also by integrated molecular diagnostics [17]. The current classification includes glioblastoma (IDH wild-type), astrocytoma IDH-mutant (WHO grade 3 or 4), oligodendroglioma (IDH-mutant and 1p/19q codeleted, WHO grade 2 or 3), and other less common molecular subtypes [32]. These new definitions carry significant implications for prognosis, treatment decisions, and clinical trial stratification, necessitating imaging tools that can not only localize and characterize tumors, but also reflect the underlying tumor biology.

MRI plays a central and evolving role throughout the continuum of glioma care. From initial detection and differential diagnosis to surgical planning, treatment response assessment, and long-term surveillance, MRI is the best imaging modality due to its superior soft tissue contrast, multiplanar capability, and growing arsenal of advanced techniques. Beyond conventional T1- and T2-weighted sequences, physiological imaging—such as diffusion-weighted imaging, perfusion-weighted imaging, magnetic resonance spectroscopy (MRS), and emerging molecular methods like amide proton transfer (APT) and 2-hydroxyglutarate (2HG) spectroscopy—offer insights into tumor biology, infiltrative behavior, and response to treatment.

## MRI techniques

### MRI perfusion

MRI perfusion refers to a group of imaging techniques designed to evaluate cerebral hemodynamics, including parameters such as cerebral blood flow (CBF), cerebral blood volume (CBV), mean transit time (MTT), and time to peak (TTP). The CBV has shown particularly relevant in the evaluation of high-grade gliomas, where neovascularization and altered vascular permeability are hallmarks of malignancy.

Three main perfusion MRI techniques are currently in clinical use: two that require gadolinium-based contrast agents, and one non-contrast-agent method. The contrast-based techniques include Dynamic Susceptibility Contrast MRI (DSC) — also referred to as T2*-weighted perfusion [7] — and Dynamic Contrast-Enhanced MRI (DCE) [48], which relies on T1-weighted imaging. The non-contrast alternative is Arterial Spin Labelling (ASL), which uses magnetically labeled arterial blood water as an endogenous tracer and is primarily used to assess CBF, although also possible to calculate CBV [47].

Each method has its distinct advantages and limitations. DSC perfusion is the most widely used and clinically validated perfusion technique. It offers high signal-to-noise ratio (SNR) and rapid acquisition, making it well-suited for estimating CBV in brain tumors. However, DSC is sensitive to susceptibility artifacts, particularly near air–bone interfaces such as the skull base, and its accuracy can be compromised in the presence of hemorrhage, calcification, or prior surgical materials, where signal loss may occur.

In such cases, DCE perfusion serves as a valuable complementary technique. DCE imaging evaluates contrast agent kinetics over time, enabling estimation of parameters such as plasma volume (Vp), vascular permeability (Ktrans), and the extracellular-extravascular space (Ve) [49]. DCE is less sensitive to susceptibility artifacts and can be useful in hemorrhagic or calcified lesions where DSC is limited. However, DCE generally suffers from a lower signal-to-noise ratio, requires more complex post-processing, and shows variation between scans [57]. Its clinical use is also less widespread than DSC, with variability in acquisition protocols and interpretation, currently limiting its standardization.

Arterial Spin Labelling (ASL) provides a non-invasive, contrast-free method for quantifying CBF and is particularly useful in patients for whom gadolinium administration is contraindicated or for other reasons withheld [1]. ASL may be beneficial in pediatric populations, patients with renal impairment, or for longitudinal studies requiring multiple time points without cumulative contrast exposure, and has shown value in differentiating between high- and low-grade gliomas [12]. However, ASL is highly sensitive to motion and susceptibility artifacts, has lower spatial resolution, and may underestimate the perfusion in regions with slow or collateral flow.

In summary, perfusion MRI is a valuable adjunct in glioma imaging, capable of demonstrating tumor vascular physiology, identifying high-grade components, and assisting in treatment planning and response assessment. The choice of technique should be guided by the clinical question, lesion characteristics, and patient-specific factors, with recognition of the technical strengths and limitations of each method.

### Diffusion weighted imaging (DWI)

Diffusion-weighted imaging (DWI) is a widely used MRI technique that measures the microscopic motion of water molecules — primarily hydrogen atoms — within tissue. This motion is influenced by several physiological factors, including cellular density, membrane integrity, extracellular space, and microstructural architecture, as well as temperature and viscosity. By incorporating diffusion-sensitizing gradients into the pulse sequence, DWI provides image contrast based on the degree of water diffusion [30].

The level of diffusion weighting in the resulting image is determined by the magnitude, duration, and timing of the applied diffusion gradients, commonly described by the b-value. Higher b-values enhance sensitivity to restricted diffusion, but may also reduce signal-to-noise ratio. In diffusion tensor imaging (DTI), which extends basic DWI to estimate the directionality of diffusion, a minimum of six non-collinear diffusion gradient directions is required to calculate the diffusion tensor [8]. This allows for the generation of white matter fiber orientation maps such as fractional anisotropy (FA) images, and fiber tractography, which are especially useful in surgical planning. The method has shown high clinical relevance but suffers from inaccuracies in the presence of for example, crossing fibers. While deterministic DTI traces the most likely single fiber pathway based on the principal diffusion direction in each voxel [40], probabilistic DTI accounts for uncertainty in fiber orientation, generating a range of possible pathways and thus capturing more complex anatomy, especially in regions with crossing fibers [2].

Building further on advanced DWI, diffusion kurtosis imaging (DKI) investigates the non gaussian distribution of water diffusion by using higher b-values and more diffusion directions compared to DTI, thereby enabling the assessment of tissue microstructure [28].

DWI is a powerful tool in the differential diagnosis of brain tumors. High-grade gliomas typically demonstrate low apparent diffusion coefficient (ADC) values in their most cellular regions, reflecting restricted diffusion due to densely packed tumor cells [51]. However, this pattern can overlap with primary CNS lymphomas, which also exhibit restricted diffusion but tend to have intermediate perfusion characteristics [51, 58] compared to highly perfused glioblastoma regions. In contrast, low-grade gliomas [54] and tumefactive demyelinating lesions [51] usually show a more facilitated diffusion, compared to the surrounding brain [19], indicative of a less dense cellular architecture. Moreover, DWI can assist in evaluating peritumoral edema, as regions with infiltrative tumor may also exhibit subtle diffusion changes compared to purely vasogenic edema [25, 37]. Diffusion kurtosis imaging has been used to differentiate between low- and high-grade gliomas [13].

Despite its diagnostic value, conventional echo planar imaging (EPI)-based DWI is susceptible to magnetic field inhomogeneities, leading to distortion artifacts near air–bone interfaces, such as the skull base or paranasal sinuses. Additionally, susceptibility effects from blood products or calcifications may degrade image quality or lead to false interpretations [29]. In these scenarios, non-EPI DWI techniques — although less commonly used in neuro-oncology — have shown utility in specific applications, such as the evaluation of cholesteatomas in the middle ear. However, its role in intracranial clinical brain tumor imaging currently remains limited [3].

In summary, DWI provides physiological information that enhances diagnostic confidence and supports surgical and oncological decision-making. Nonetheless, its limitations in certain anatomical regions and pathological conditions must be considered, and integration with perfusion and conventional imaging is often required for an accurate interpretation.

### MR spectroscopy

Magnetic resonance spectroscopy (MRS) is an advanced imaging technique that allows for the non-invasive quantification of metabolites within brain tissue. Depending on the acquisition protocol, MRS can be performed in a single small region (single voxel) or across larger areas of the brain (multivoxel or whole-brain spectroscopy). The main limitations of whole-brain MRS remain prolonged acquisition times and lower spatial resolution, which can affect clinical feasibility.

Several MRS sequences have been developed to optimize signal acquisition, the most commonly used being PRESS (Point RESolved Spectroscopy), STEAM (Stimulated Echo Acquisition Mode), with a more recent addition of sLASER (semi-Localized by Adiabatic Selective Refocusing). Each has its own balance of signal-to-noise ratio, spatial resolution, and sensitivity to artifacts. In clinical settings, and due to software constraints, the focus is often on the metabolic profile of the tissue, and on metabolite ratios rather than absolute quantification, which is more technically challenging.

In brain tumor imaging, multivoxel MRS provides a more detailed view of the metabolic heterogeneity of lesions. High-grade gliomas typically show elevated choline levels, reflecting increased membrane turnover and cellular proliferation, along with a reduction in N-acetylaspartate (NAA), a marker of neuronal integrity. Additional spectral peaks, such as lactate and lipid macromolecules, may appear in necrotic tumor cores, further supporting the diagnosis [21].

MRS can also be helpful in differentiating tumor types. For instance, in ring-enhancing lesions, the peritumoral zone in glioblastomas often contains infiltrative tumor cells that alter the metabolic landscape beyond the contrast-enhancing margin. This is reflected by abnormal metabolite ratios on MRS, such as an elevated choline-to-NAA ratio, extending into the surrounding tissue. In contrast, metastases generally cause vasogenic edema in the peritumoral region, which typically appears metabolically normal on spectroscopy [20]. This distinction can be crucial in both diagnosis and surgical planning.

While MRS adds important physiological information, the technique also has limitations. Spatial resolution is relatively low, and voxel placement is critical to avoid partial volume effects from CSF, necrotic, and even from normal tissue. MRS is also sensitive to motion artifacts, magnetic field inhomogeneities, as well as from fat signal contamination or signal “bleed” from the skull. Interpretation of spectral data requires specialized expertise, and standardization across vendors remains limited, which can affect reproducibility and clinical implementation.

In summary, MR spectroscopy is a valuable tool for characterizing brain tumors beyond their morphological appearance, especially when used as part of a multimodal MRI protocol. It can guide biopsies, affirm surgical margins, and support the differentiation of glioblastoma from non-infiltrative lesions, although technical and interpretive challenges continue to limit its widespread routine use.

## MRI at presentation

High-grade gliomas (HGGs) are rarely incidental findings. Rather, they typically manifest with acute or subacute neurological symptoms that prompt urgent neuroimaging. Presenting complaints may include seizures, progressive headaches, focal neurological deficits, cognitive changes, or signs of increased intracranial pressure such as nausea, vomiting, and papilledema [34, 41].

The relatively short symptom duration preceding diagnosis reflects the tumor’s high mitotic rate and aggressive biological behavior, distinguishing HGGs from indolent brain lesions [32]. In these situations, brain MRI, including advanced sequences, leads the patient to a preliminary radiologic diagnosis of a high-grade glioma based on either the presentation of a tumor with ring-like contrast enhancement and necrotic core, or a diffusely infiltrative tumor on T2-FLAIR but with high perfusion (cerebral blood volume on dynamic susceptibility imaging) or low diffusion [17].

At the time of diagnosis, one of the most important roles of MRI is to help distinguish true brain tumors from other conditions that can appear similar on imaging. This distinction is crucial—especially before any surgical intervention—since some lesions may instead represent treatable non-neoplastic conditions such as neuroinflammation [9, 55] or infection [5]. It's essential to ensure that patients considered for tumor resection actually have a brain tumor, to avoid unnecessary surgery.

Although advanced MRI techniques have improved our ability to make these distinctions, some cases remain challenging. When doubt persists, a targeted biopsy can provide a definitive diagnosis and is especially useful in ambiguous or atypical presentations. Still, certain advanced imaging sequences can offer helpful clues to the right diagnosis.

**Fig. 1:**
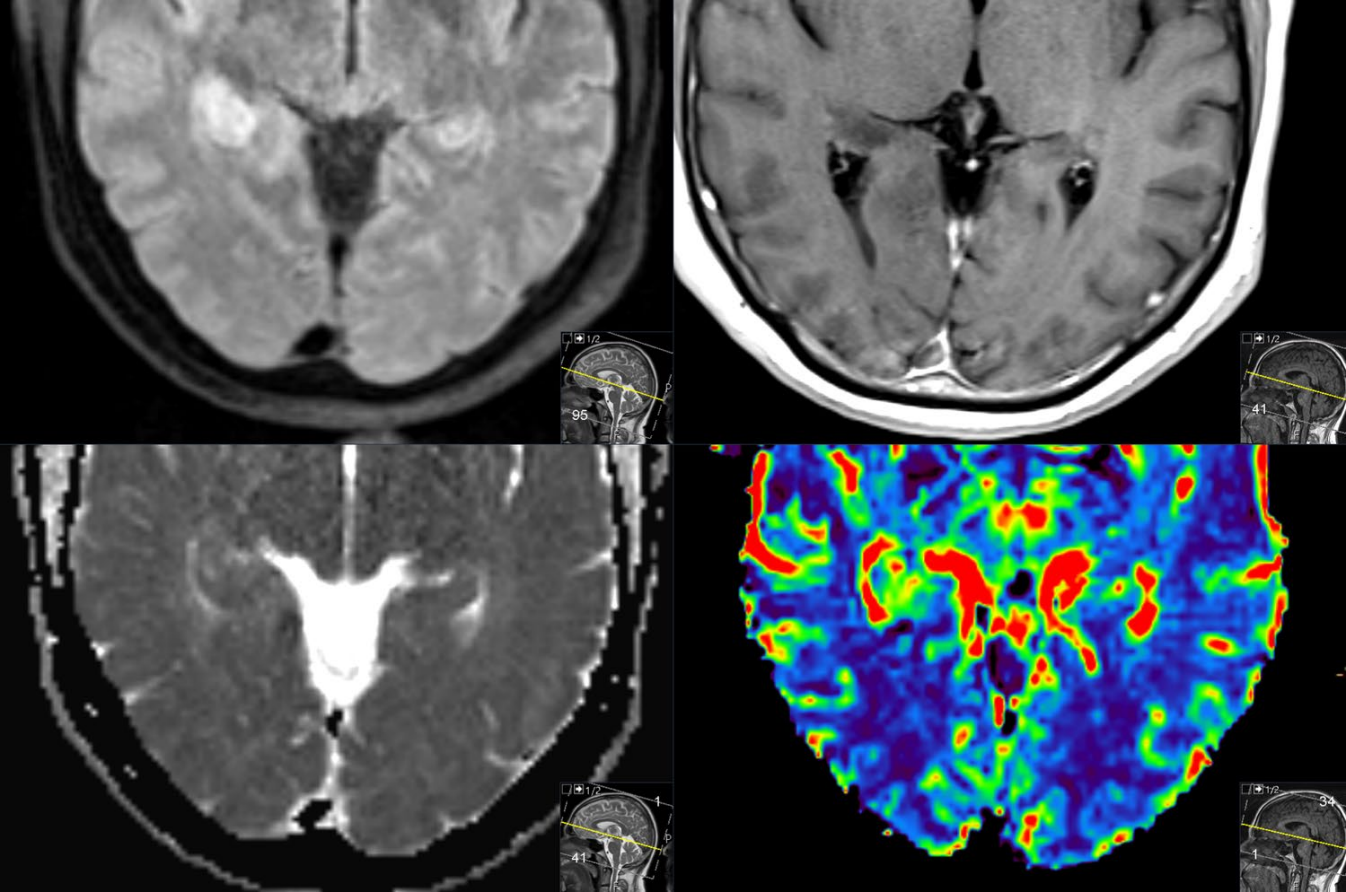
Patient with a molecular glioblastoma (histopathological confirmation, IDH wild type). Upper left, DWI with high signal intensity, lower left, ADC map with isointensity. Upper right, T1 post contrast without enhancement, lower right, dynamic susceptibility perfusion MRI with high cerebral blood volume

MR perfusion imaging can, by measuring the cerebral blood volume, help to identify areas of neovascularization, an important feature of high-grade gliomas. Further, diffusion-weighted imaging can detect areas with restricted or low diffusion, which often reflects the high cellularity typical of these tumors. When used together, these techniques can increase the diagnostic confidence and help guide management decisions before surgery [15], Fig. 1.

**Fig. 2:**
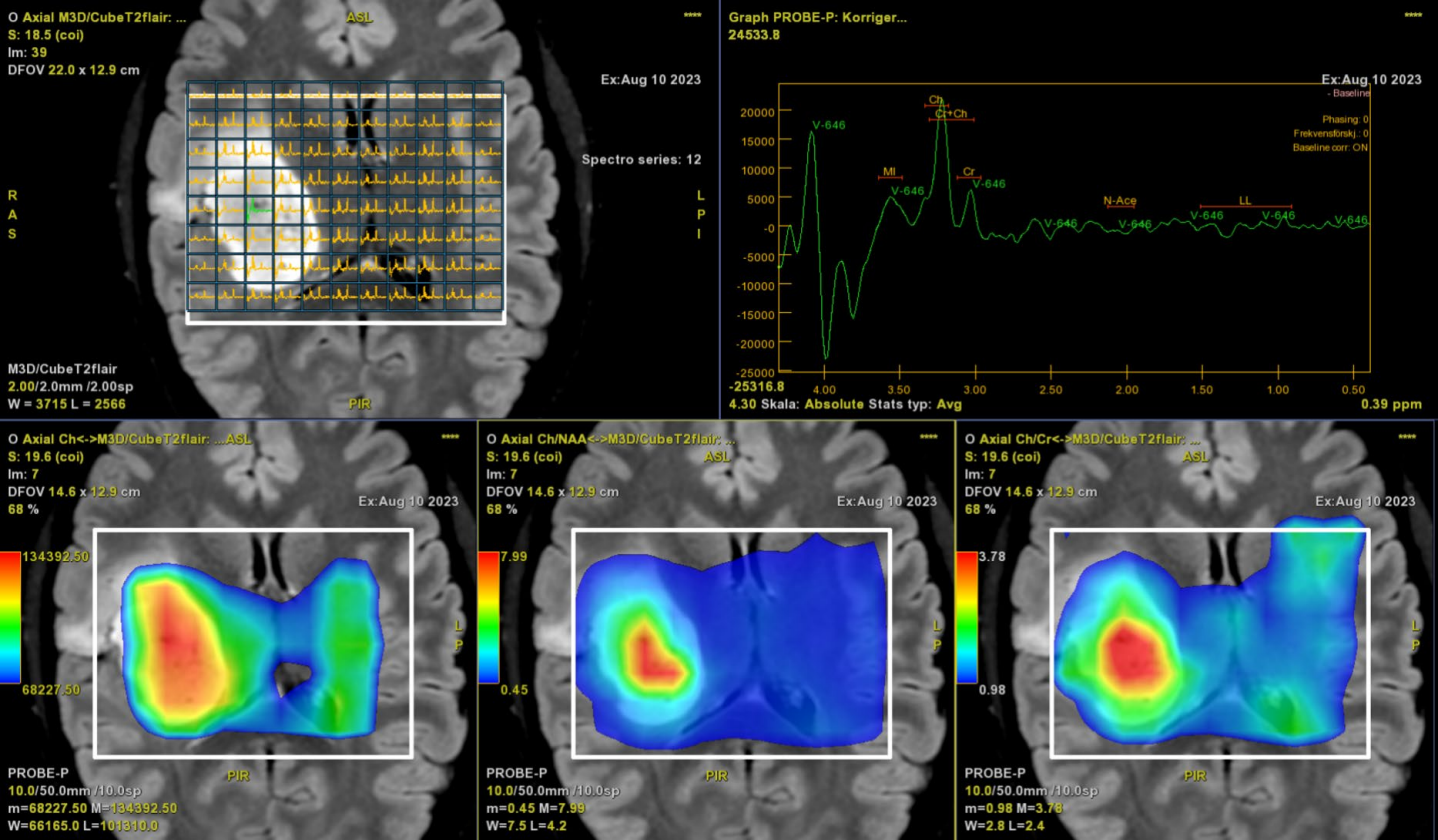
Patient with an astrocytoma grade 4 (IDH-mutated). MR multivoxel spectroscopy with high choline and low NAA inside the tumor. Metabolic ratios Choline/NAA and Choline/Creatin are also high

Moreover, it is also preferred if imaging can triage patients with high-grade tumors to subacute surgery, as opposed to patients with low-grade tumors that can be managed in a non-emergent setting [27]. Imaging can also aid by indicating the need for further diagnostic work-up in patients with suspected metastasis from a non-CNS primary as metastases have a less infiltrative growth pattern compared to glioblastomas. In addition to standard T1 with gadolinium and T2/T2FLAIR weighted images, other more advanced MR imaging techniques, such as perfusion, diffusion, and spectroscopy, can be helpful for a complete assessment of suspected tumor type and biologic behavior before surgery, as well as direct focus biopsies towards areas of maximum pathology, Fig. 2.

Importantly, even with advanced imaging, there remains a degree of diagnostic uncertainty — particularly in cases with atypical imaging features or when distinguishing tumor from inflammatory mimics. Imaging findings should therefore always be interpreted in conjunction with clinical presentation and, when appropriate, supplemented with histopathologic confirmation through biopsy. In summary, while advanced MRI techniques significantly enhance preoperative assessment they do not replace tissue diagnosis and should be viewed as complementary tools that can improve — but not eliminate — the inherent uncertainty in tumor characterization.

## Preoperative MRI

Preoperative MRI is essential in the management of high-grade gliomas (HGGs), as it provides the neurosurgeon with critical information regarding tumor location, extent, and its relationship to eloquent brain regions. The overarching goal is to enable maximal safe resection — removing as much tumor as possible while preserving neurological function. Advanced MRI techniques have significantly expanded the scope of preoperative evaluation, allowing for more precise surgical planning and risk assessment.

**Fig. 3:**
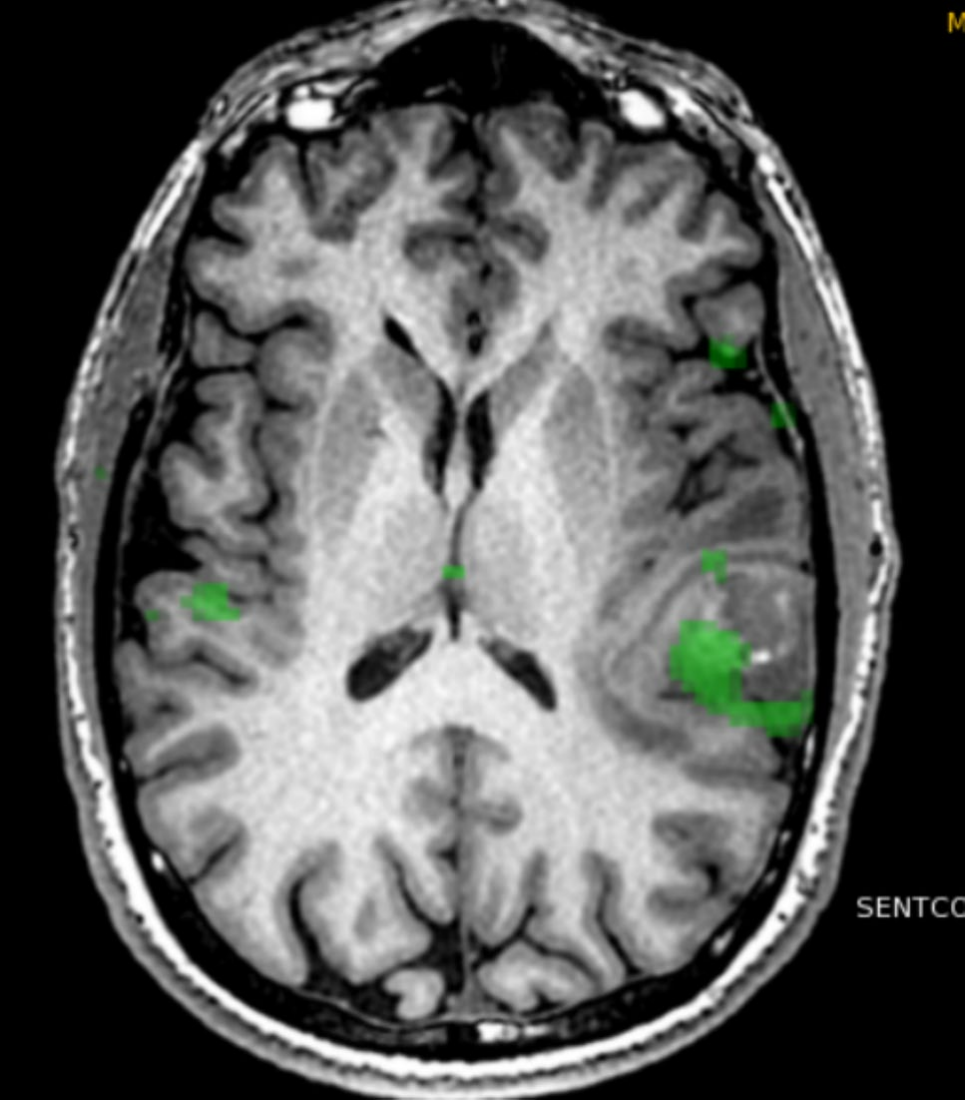
Patient with a glioblastoma in the left temporal lobe. Functional MRI depicting left sided language lateralization

One of the most clinically established techniques in this setting is functional MRI (fMRI), which leverages Blood Oxygen Level Dependent (BOLD) contrast to detect signal changes linked to variations in the local ratio of deoxyhemoglobin and oxyhemoglobin during neural activity. This method allows for localization and lateralization of eloquent cortical areas, including those involved in language and motor functions, by having the patient perform task-based paradigms during scanning [18, 26]. In the context of tumors near or within eloquent regions, such as the primary motor cortex or Broca’s area, this information is important for optimizing safe resection margins (see Fig. 3).

However, fMRI also has inherent limitations. It is highly dependent on patient cooperation and optimal task performance, which may be compromised in patients with neurological deficits. Moreover, the BOLD signal is an indirect marker of neuronal activity and can be distorted by neurovascular uncoupling in and around gliomas, leading to false-negative or spatially inaccurate activations. To improve accuracy and contextualize fMRI findings, it is often integrated with diffusion tensor imaging (DTI).

**Fig. 4:**
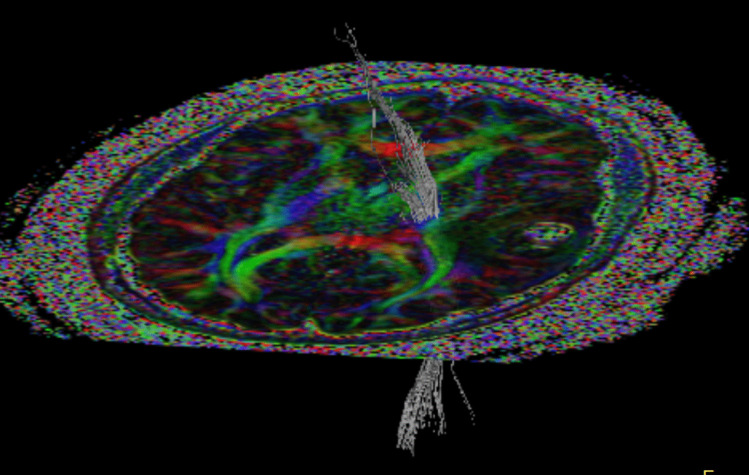
Patient with a glioblastoma (same as in Fig. 3) with the corticospinal tract depicted from MR tractography located medial to the tumor in the left temporal lobe

As previously mentioned, DTI provides visualization of white matter tracts by mapping the directionality of water diffusion in brain tissue. By reconstructing the primary diffusion vectors across voxels, DTI allows for tractography — a probabilistic representation of thick fiber bundles such as the corticospinal tract or arcuate fasciculus (see Fig. 4). When combined with fMRI-derived cortical activation maps, DTI enables a three-dimensional assessment of both functional and structural connectivity, facilitating the identification of “safe corridors” and helping to minimize the risk of motor or language deficits postoperatively [31].

Nevertheless, DTI also faces technical challenges. It can be sensitive to distortion artifacts, crossing fibers, and tumor-related displacement or infiltration of tracts [11], all of which may impact accuracy. Probabilistic methods and high-angular resolution diffusion imaging (HARDI) are being developed to improve upon these limitations, but are not yet widely adopted clinically.

Beyond functional and structural localization, understanding the biological and metabolic landscape of tumor infiltration has become increasingly important for planning extended or supratotal resections. Conventional contrast-enhanced T1-weighted MRI delineates the blood–brain barrier breakdown but underestimates the true extent of glioma infiltration, which may extend beyond the enhancing margin. Advanced techniques such as magnetic resonance spectroscopy (MRS), amide proton transfer (APT) imaging, perfusion, and diffusion MRI can provide complementary information on tumor biology.

As previously mentioned, whole-brain or 3D chemical shift imaging (CSI) allows for the mapping of tissue metabolite concentrations across the brain. A well-established metabolic marker is the choline-to-N-acetylaspartate (Cho/NAA) ratio, which is often elevated in glioblastoma tissue — even in non-enhancing, infiltrative zones [20, 43]. Identifying such areas may guide the extent of resection in patients eligible for more extensive surgical approaches.

Similarly, APT imaging, a subtype of chemical exchange saturation transfer (CEST) imaging, detects endogenous amide protons that exchange with water, providing contrast based on protein and peptide content. This technique has been shown to discriminate high-grade from low-grade gliomas [45] and to highlight tumor infiltration beyond the contrast-enhancing border [46, 52]. Though still investigational, APT may evolve into a valuable tool for preoperative tumor margin assessment.

Additional insights can be obtained from perfusion-weighted imaging, particularly dynamic susceptibility contrast (DSC) MRI, which maps cerebral blood volume (CBV). Higher CBV in peritumoral regions compared to in perilesional edema without tumor infiltration, may reflect angiogenesis and thus viable tumor infiltration, while diffusion MRI can highlight increased cellularity through reduced apparent diffusion coefficient (ADC) values — both providing a functional map of potentially infiltrated brain tissue not evident on conventional T2-FLAIR or T1-weighted sequences [53].

In summary, preoperative MRI in high-grade gliomas extends also beyond conventional structural imaging, incorporating a growing set of advanced techniques that provide functional, metabolic, and physiological insights into brain and tumor architecture and function. These tools may enhance surgical planning by offering a more detailed understanding of tumor infiltration and eloquent area involvement. However, although promising, these techniques are not without limitations. Their successful clinical use depends not only on optimal technical execution, but also on expert interpretation, full patient cooperation, and reflective integration into the broader clinical and surgical context.

It is important to acknowledge that many of these methods — including fMRI, DTI, MRS, APT, and perfusion imaging — still face challenges related to standardization, susceptibility to artifacts, spatial accuracy, and availability across institutions. Interpretation is often non-binary and nuanced, requiring correlation with conventional imaging and clinical context. In certain scenarios, these modalities may overestimate or underestimate tumor extent or eloquence, and caution should be exercised in applying them directly to intraoperative decision-making without multidisciplinary review.

Ultimately, advanced MRI should be seen not as a definitive answer but as a supportive tool — one that complements the neurosurgeon’s experience and intraoperative judgment. As techniques continue to mature and as integration into neuronavigation platforms improves, these modalities are likely to play an increasingly important role in personalized and precise surgical strategies in the future. Continued dialogue between neuroradiologists and neurosurgeons will be essential to refine the value, reliability, and clinical integration of these imaging approaches.

## Intraoperative MRI

During surgery, preoperative MRI can be integrated into the neuronavigation system, allowing the neurosurgeon to align surgical entry and trajectory with the planned resection corridor, while maintaining spatial awareness of nearby eloquent cortical areas and white matter tracts [6, 36] and other important structures such as vessels and nerves. This neuronavigational approach may improve surgical orientation and it also may enhance the precision of tumor targeting, particularly in irregularly shaped or deep-seated lesions where direct vision is reduced.

However, a common limitation of relying solely on preoperative images is the phenomenon of brain shift. When the dura is opened and the cerebrospinal fluid is released, the brain may deform significantly due to gravity, edema, resection, or manipulation. As a result, the preoperative MRI becomes an increasingly less accurate map as the procedure progresses. To compensate for this, intraoperative MRI (iMRI) has been introduced, allowing for repeat imaging during surgery to account for changes in brain morphology and to update neuronavigation accordingly [14, 22, 36]. When performed after resection, iMRI can reveal residual tumor tissue, enabling immediate re-intervention during the same procedure. This may reduce the need for repeat surgeries prompted by unexpected findings on postoperative MRI, typically acquired within 48–72 h after the initial operation [4]. iMRI has also been shown to be more accurate compared to early postoperative MRI [39].

Despite these benefits, several practical and technical challenges must be recognized. First, the setup and operation of an iMRI suite involves a considerable infrastructure investment. It requires specially designed operating rooms that are MRI-compatible, along with strict adherence to safety protocols for ferromagnetic equipment and monitoring devices. Patient positioning must be carefully maintained between surgical steps and imaging, which can prolong operative time and potentially impact workflow efficiency.

Additionally, interrupting surgery for scanning — including sterile draping, re-sterilization after imaging, and system recalibration — can be time-consuming. In some settings, this extended operative time may increase anesthesia-related risks or strain operating room resources. Artifacts from blood, air, or hardware can compromise image quality, and interpretation of intraoperative images — particularly in distinguishing residual tumor from surgically induced changes such as edema or hemorrhage — can be challenging.

There is also the human factor: iMRI interpretation during surgery places time pressure on radiologists or surgeons reviewing the images, which may lead to more conservative reads. Furthermore, the presence of intraoperative MR images does not guarantee better outcomes unless findings are effectively acted upon in real time. In complex cases, imaging feedback may alter the surgical plan in ways that require careful multidisciplinary discussion, which is not always feasible intraoperatively.

In summary, intraoperative MRI can enhance surgical precision, reduce reoperation rates, and offer real-time assessment of resection extent. However, its logistical complexity, cost, potential for workflow disruption, and interpretive challenges limit its widespread use. Its value is likely greatest in high-risk or high-value resections, such as those near eloquent cortex or where maximizing resection correlates strongly with prognosis. As with all advanced imaging tools, iMRI is most effective when applied selectively, in close collaboration between neurosurgery, anesthesiology, and neuroradiology.

## Postoperative MRI

Postoperative MRI plays a central role in assessing residual tumor, detecting surgical complications, and informing further treatment planning. When performed within 48–72 h after surgery, it provides the clearest view of residual contrast-enhancing tumor before postoperative changes—such as inflammation or blood–brain barrier disruption—begin to blur interpretation [44].

Standard postoperative protocols typically include contrast-enhanced T1-weighted sequences to identify residual tumor, T2-weighted or susceptibility-weighted imaging (SWI) to evaluate hemorrhage, and diffusion-weighted imaging (DWI) to detect ischemia or infarcts. These MRI sequences often replace the need for a postoperative CT and are essential for both early complication detection and confirmation of resection extent.

More advanced MRI techniques—such as spectroscopy, perfusion imaging, or advanced diffusion sequences—are usually not included at this early stage. Their use is often limited by practical considerations such as patient condition, susceptibility to artifacts (e.g., from air or blood products), and the relatively limited added diagnostic value immediately postoperatively. However, these methods may be valuable later during surveillance or re-treatment planning.

For neurosurgeons, the early postoperative MRI provides critical feedback on the extent of resection and complements intraoperative assessments. Yet interpretation must be cautious—enhancement along the resection margin can represent residual tumor, reactive postoperative changes, or vascular congestion, depending in part on timing. Close communication between the radiologist and surgical team is key to accurate interpretation and optimal patient care.

## Radiation therapy MRI

Importantly, the immediate postoperative MRI is not typically used for radiotherapy planning, as the surgical cavity and enhancement patterns continue to resolve for several weeks. A separate, delayed planning scan is usually acquired once the postoperative anatomy has stabilized, and within 2 weeks from radiotherapy start [36]. 

Historically, radiotherapy planning for high-grade gliomas has relied on computed tomography (CT) with intravenous contrast for delineation of treatment volumes. However, current guidelines for radiation therapy planning for glioblastoma incorporates MRI into the radiotherapy planning workflow, particularly as more cancer centers adopt in-house MR imaging capabilities [38].

In current clinical practice, conventional MRI sequences, primarily T1-weighted post-gadolinium and T2/FLAIR, are used to define gross tumor volume (GTV) and surrounding edema, respectively. These sequences offer superior soft-tissue contrast compared to CT and better visualization of residual enhancing tumor, surgical cavity boundaries, and infiltrative edema — all critical in determining clinical target volumes (CTV) and planning treatment margins.

However, gliomas often extend microscopically beyond the visible margins on anatomical MRI. Consequently, there is increasing interest in advanced MRI techniques that can offer biological or functional guidance for radiotherapy — with the aim of moving from uniform dose delivery to dose painting strategies, where radiation is intensified to the most aggressive tumor regions while sparing surrounding tissue [38, 42].

Perfusion imaging, particularly dynamic susceptibility contrast (DSC) MRI, may identify hyperperfused areas indicative of angiogenesis and higher tumor activity. Similarly, diffusion-weighted imaging (DWI) and ADC maps can highlight regions of increased cellularity, and MR spectroscopy can detect metabolic abnormalities such as elevated choline-to-NAA ratios. These physiologic markers may better reflect viable tumor burden than contrast enhancement alone.

While the clinical implementation of these techniques remains limited by standardization issues, variable reproducibility, and complex integration into radiotherapy planning systems, research is ongoing to validate their predictive value and to develop robust, automated workflows. Some institutions have started pilot programs using biologically guided radiotherapy, integrating maps of perfusion or metabolism into treatment planning software to allow dose escalation to high-risk subvolumes [24].

In the near term, conventional MRI sequences will likely remain the foundation of radiotherapy planning, but advanced imaging may increasingly serve as a complementary tool — particularly in selected cases where tumor heterogeneity, unusual enhancement patterns, or treatment resistance is suspected. Collaboration between neuroradiologists, radiation oncologists, medical and MRI physicists is essential to move new techniques from research applications to reliable clinical tools.

In summary, MRI in radiotherapy planning is currently evolving from a purely anatomical roadmap to a potential biological guide, supporting precision oncology approaches. As evidence grows and software integration improves, advanced MRI could become central not only to visualizing where the tumor is — but understanding where it is most active, and potentially most lethal.

## Treatment surveillance

MRI plays an important role during disease surveillance by aiding in the differentiation between true tumor progression and treatment-related changes and assessing treatment response. For example, treatment-related changes such as pseudoprogression and radiation necrosis will show lower cerebral blood volume (CBV) compared to true tumor progression [10], indicating the important role of neovascularization for viable tumor and vessel impairment through hyalinization and coagulation necrosis with contrast agent leakage without perfusion increase in treatment-related changes [35]. However, this assessment is often complicated by the two states occurring concurrently, either in the same regions or in different areas of the treated tumor. The complexity in this radiological evaluation, also taking into account the clinical status of the patient, current treatment therapies and possible ongoing clinical studies necessitates a discussion of these patients at multidisciplinary tumor rounds. 

Further, previously mentioned techniques used to differentiate high-grade gliomas from low-grade can also be used to differentiate tumor progression from treatment-related changes that, despite their different pathophysiology, will appear similar to each other on contrast-enhanced T1-weighted images. An important role in treatment surveillance is deciding when the tumor is progressing in a way that would necessitate reconsidering current treatment. A tumor progression during follow-up might require changing chemotherapy, reconsidering surgery or radiation therapy, adding localized microsurgical techniques such as laser-induced thermal therapy (LITT) or gamma knife surgery (GNS), or directing the patient to off-label treatments or the inclusion into clinical trials investigating new treatments such as immunotherapy.

In treatment surveillance for follow-up of patients with high-grade glioma, imaging has to be both sensitive enough to catch new small lesions accessible for localized treatment. At the same time, it has to be highly specific and indicate true tumor progression correctly before re-operation and chemotherapy changes are considered. High-resolution 3D imaging is performed with T1 weighted images after gadolinium injection and T2-FLAIR images to increase sensitivity. To increase specificity, the physiological properties of the tissue have to be investigated, with perfusion and diffusion [23], sometimes complemented with MRI spectroscopy [16, 33, 43] or other advanced imaging techniques such as PET-imaging. Another standardized way of doing this in clinical trials is assessing tumor size increase on T1 weighted images after gadolinium in contrast-enhancing high-grade glioma, as advocated by the Response Assessment in Nero-Oncology [56].

**Fig. 5:**
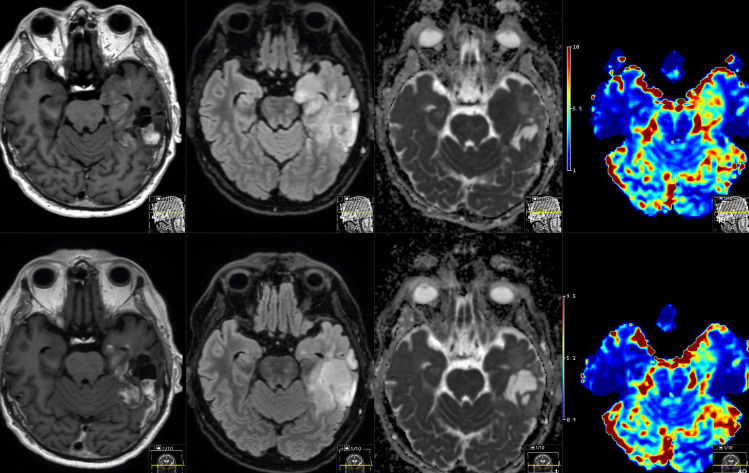
Patient with a glioblastoma investigated during treatment surveillance and Bevacizumab treatment. Lower row, before Bevacizumab treatment, and upper row after Bevacizumab treatment. After treatment, the contrast enhancement is slightly less pronounced at T1 post contrast images (left column), while the T2-FLAIR signal changes still continues to progress (second column from the left), the ADC-signal is not facilitated as would be expected in areas with less cell density, and the perfusion images shows that there still is hyperperfused progressing tumor areas

Although not yet widely implemented clinically, future automatic brain tumor segmentation with RANO assessment techniques, would make it possible to include it in a standardized radiology report, complementing the radiologist's assessment. A caveat today to the standardized evaluation of only contrast-enhancing area or volume is that it needs to be adapted when, for example, antiangiogenetic treatment is used, such as VEGF inhibitors, and loss of contrast enhancement is an expected feature. However, tumor size can grow on T2FLAIR or DWI images during anti VEGF treatment, Fig. 5.

Another more complex question is how to radiologically assess the proportion of viable tumor in an area with radiation necrosis. This important question requires further investigation, and new MRI methods must be continually developed to enhance the diagnostic power of imaging. Looking further into the future of brain tumor imaging, with genetic profiling and molecular tumor traits, future MRI methods might aim to reflect these tumor properties. An emerging technique with direct molecular MRI profiling is 2HG spectroscopy, where 2HG—an oncometabolite in IDH-mutated gliomas—can be quantified in brain tissue imaging, and its distribution in the tissue can be assessed [50] — a potentially important topic in an area with new emerging drugs such as IDH-inhibitors.

## The future of MRI in high-grade gliomas

Ongoing developments in MRI technology, including higher magnetic field strengths, continue to improve image resolution, signal quality, and shorten scan times. In parallel, artificial intelligence and machine learning are gradually being integrated into clinical workflows to enhance image acquisition, reconstruction, and postprocessing—making advanced imaging more accessible and consistent.

As new treatment strategies emerge—such as personalized gene therapies, precision oncology, and immunotherapy—there is a growing need for imaging to provide more than just structural information. These therapies often target molecular features, which highlights the potential role of advanced MRI techniques in assessing the metabolic and physiological properties of high-grade gliomas.

Progress in this area will depend not only on continued technical improvements in MRI hardware and software, but also on close collaboration between neuroradiologists, neurosurgeons, and neuro-oncologists. By working together, we can better tailor imaging to evolving clinical needs and contribute to improved outcomes and quality of life for patients.

## Data Availability

No datasets were generated or analysed during the current study.

## References

[1] Alsop DC, Detre JA, Golay X et al (2015) Recommended implementation of arterial spin-labeled perfusion MRI for clinical applications: a consensus of the ISMRM perfusion study group and the European consortium for ASL in dementia. Magn Reson Med 73(1):102–116 24715426 10.1002/mrm.25197 PMC4190138

[2] Behrens TEJ, Berg HJ, Jbabdi S, Rushworth MFS, Woolrich MW (2007) Probabilistic diffusion tractography with multiple fibre orientations: what can we gain? Neuroimage 34(1):144–155 17070705 10.1016/j.neuroimage.2006.09.018 PMC7116582

[3] Benson JC, Carlson ML, Lane JI (2021) Non-EPI versus multishot EPI DWI in cholesteatoma detection: correlation with operative findings. AJNR Am J Neuroradiol 42(3):573–577 33334855 10.3174/ajnr.A6911 PMC7959419

[4] Bette S, Gempt J, Huber T, Boeckh-Behrens T, Ringel F, Meyer B, Zimmer C, Kirschke JS (2016) Patterns and time dependence of unspecific enhancement in postoperative magnetic resonance imaging after glioblastoma resection. World Neurosurg 90:440–447 27001238 10.1016/j.wneu.2016.03.031

[5] Boa Sorte AA Jr, Garcia CC, Neto MR, de Oliveira MF, Rotta JM (2022) Brain cryptococcoma mimicking a glioblastoma in an immunocompetent patient: a rare case report and comprehensive review. SurgNeurol Int 13:114. 10.25259/SNI_1243_2021 10.25259/SNI_1243_2021 PMC9062938 35509529

[6] Bonosi L, Marrone S, Benigno UE, Buscemi F, Musso S, Porzio M, Silven MP, Torregrossa F, Grasso G (2023) Maximal safe resection in glioblastoma surgery: a systematic review of advanced intraoperative image-guided techniques. Brain Sci. 10.3390/brainsci13020216 36831759 10.3390/brainsci13020216 PMC9954589

[7] Boxerman JL, Quarles CC, Hu LS et al (2020) Consensus recommendations for a dynamic susceptibility contrast MRI protocol for use in high-grade gliomas. Neuro Oncol 22(9):1262–1275 32516388 10.1093/neuonc/noaa141 PMC7523451

[8] Campbell JSW, Siddiqi K, Rymar VV, Sadikot AF, Pike GB (2005) Flow-based fiber tracking with diffusion tensor and q-ball data: validation and comparison to principal diffusion direction techniques. Neuroimage 27(4):725–736 16111897 10.1016/j.neuroimage.2005.05.014

[9] Cereda GS, Doniselli FM, Deleo F et al (2025) High-grade gliomas with autoimmune encephalitis-like presentation: case report and systematic review of the literature. Neurol Sci. 10.1007/s10072-025-08159-x 40214926 10.1007/s10072-025-08159-x

[10] Chen H, Tan G, Zhong L et al (2024) MR perfusion characteristics of pseudoprogression in brain tumors treated with immunotherapy - a comparative study with chemo-radiation induced pseudoprogression and radiation necrosis. J Neurooncol. 10.1007/s11060-024-04910-0 39688766 10.1007/s11060-024-04910-0 PMC11832695

[11] Delgado AF, Nilsson M, Latini F, Mårtensson J, Zetterling M, Berntsson SG, Alafuzoff I, Lätt J, Larsson E-M (2016) Preoperative quantitative MR tractography compared with visual tract evaluation in patients with neuropathologically confirmed gliomas grades II and III: a prospective cohort study. Radiol Res Pract 2016(1):7671854 27190647 10.1155/2016/7671854 PMC4852118

[12] Falk Delgado A, De Luca F, van Westen D, Falk Delgado A (2018) Arterial spin labeling MR imaging for differentiation between high- and low-grade glioma-a meta-analysis. Neuro Oncol 20(11):1450–1461 29868920 10.1093/neuonc/noy095 PMC6176798

[13] Falk Delgado A, Nilsson M, van Westen D, Falk Delgado A (2018) Glioma grade discrimination with MR diffusion kurtosis imaging: a meta-analysis of diagnostic accuracy. Radiology 287(1):119–127 29206593 10.1148/radiol.2017171315

[14] Fan X, Roberts DW, Schaewe TJ, Ji S, Holton LH, Simon DA, Paulsen KD (2017) Intraoperative image updating for brain shift following dural opening. J Neurosurg 126(6):1924–1933 27611206 10.3171/2016.6.JNS152953 PMC5549265

[15] Farace P, Amelio D, Ricciardi GK, Zoccatelli G, Magon S, Pizzini F, Alessandrini F, Sbarbati A, Amichetti M, Beltramello A (2013) Early MRI changes in glioblastoma in the period between surgery and adjuvant therapy. J Neurooncol 111(2):177–185 23264191 10.1007/s11060-012-0997-y

[16] Feng A, Yuan P, Huang T, Li L, Lyu J (2022) Distinguishing tumor recurrence from radiation necrosis in treated glioblastoma using multiparametric MRI. Acad Radiol 29(9):1320–1331 34896001 10.1016/j.acra.2021.11.008

[17] Foltyn-Dumitru M, Banan R, Schell M, Mahmutoglu MA, Kessler T, Wick W, Brugnara G, Bendszus M, Sahm F, Vollmuth P (2025) Histopathological and molecular characteristics of IDH-wildtype glioblastoma without contrast enhancement: implications for clinical outcomes. Neuro Oncol. 10.1093/neuonc/noaf070 40108725 10.1093/neuonc/noaf070 PMC12417822

[18] Gabriel M, Brennan NP, Peck KK, Holodny AI (2014) Blood oxygen level dependent functional magnetic resonance imaging for presurgical planning. Neuroimaging Clin N Am 24(4):557–571 25441500 10.1016/j.nic.2014.07.003

[19] Hiremath SB, Muraleedharan A, Kumar S, Nagesh C, Kesavadas C, Abraham M, Kapilamoorthy TR, Thomas B (2017) Combining diffusion tensor metrics and DSC perfusion imaging: can it improve the diagnostic accuracy in differentiating tumefactive demyelination from high-grade glioma? AJNR Am J Neuroradiol 38(4):685–690 28209583 10.3174/ajnr.A5089 PMC7960259

[20] Hung ND, Van Dung L, Vi NH, Hai Anh N-T, Hong Phuong L-T, Hieu ND, Duc NM (2023) The role of 3-Tesla magnetic resonance perfusion and spectroscopy in distinguishing glioblastoma from solitary brain metastasis. J Clin Imaging Sci 13:19 37559877 10.25259/JCIS_49_2023 PMC10408633

[21] Ikeguchi R, Shimizu Y, Abe K, Shimizu S, Maruyama T, Nitta M, Abe K, Kawamata T, Kitagawa K (2018) Proton magnetic resonance spectroscopy differentiates tumefactive demyelinating lesions from gliomas. Mult Scler Relat Disord 26:77–84 30237108 10.1016/j.msard.2018.08.025

[22] Iversen DH, Wein W, Lindseth F, Unsgård G, Reinertsen I (2018) Automatic intraoperative correction of brain shift for accurate neuronavigation. World Neurosurg 120:e1071–e1078 30213682 10.1016/j.wneu.2018.09.012

[23] Kim HS, Goh MJ, Kim N, Choi CG, Kim SJ, Kim JH (2014) Which combination of MR imaging modalities is best for predicting recurrent glioblastoma? Study of diagnostic accuracy and reproducibility. Radiology 273(3):831–843 24885857 10.1148/radiol.14132868

[24] Kim MM, Parmar HA, Aryal MP, Mayo CS, Balter JM, Lawrence TS, Cao Y (2019) Developing a pipeline for multiparametric MRI-guided radiation therapy: initial results from a phase II clinical trial in newly diagnosed glioblastoma. Tomography 5(1):118–126 30854449 10.18383/j.tom.2018.00035 PMC6403045

[25] Kolakshyapati M, Adhikari RB, Karlowee V et al (2018) Nonenhancing peritumoral hyperintense lesion on diffusion-weighted imaging in glioblastoma: a novel diagnostic and specific prognostic indicator. J Neurosurg 128(3):667–678 28362236 10.3171/2016.10.JNS161694

[26] Krings T, Schreckenberger M, Rohde V et al (2002) Functional MRI and 18F FDG-positron emission tomography for presurgical planning: comparison with electrical cortical stimulation. Acta Neurochir (Wien) 144(9):889–99 (discussion 899) 12376770 10.1007/s00701-002-0992-8

[27] Law M, Young RJ, Babb JS, Peccerelli N, Chheang S, Gruber ML, Miller DC, Golfinos JG, Zagzag D, Johnson G (2008) Gliomas: predicting time to progression or survival with cerebral blood volume measurements at dynamic susceptibility-weighted contrast-enhanced perfusion MR imaging. Radiology 247(2):490–498 18349315 10.1148/radiol.2472070898 PMC3774106

[28] Lazar M, Jensen JH, Xuan L, Helpern JA (2008) Estimation of the orientation distribution function from diffusional kurtosis imaging. Magn Reson Med 60(4):774–781 18816827 10.1002/mrm.21725 PMC2562250

[29] Le Bihan D, Poupon C, Amadon A, Lethimonnier F (2006) Artifacts and pitfalls in diffusion MRI. J Magn Reson Imaging 24(3):478–488 16897692 10.1002/jmri.20683

[30] Le Bihan D, Turner R, Douek P, Patronas N (1992) Diffusion MR imaging: clinical applications. AJR Am J Roentgenol 159(3):591–599 1503032 10.2214/ajr.159.3.1503032

[31] Lolli VE, Coolen T, Sadeghi N, Voordecker P, Lefranc F (2023) BOLD fMRI and DTI fiber tracking for preoperative mapping of eloquent cerebral regions in brain tumor patients: impact on surgical approach and outcome. Neurol Sci 44(8):2903–2914 36914833 10.1007/s10072-023-06667-2

[32] Louis DN, Perry A, Wesseling P et al (2021) The 2021 WHO classification of tumors of the central nervous system: a summary. Neuro Oncol 23(8):1231–1251 34185076 10.1093/neuonc/noab106 PMC8328013

[33] Lu W, Feng J, Zou Y, Liu Y, Gao P, Zhao Y, Wu X, Ma H (2024) 1H-MRS parameters in non-enhancing peritumoral regions can predict the recurrence of glioblastoma. Sci Rep 14(1):29258 39587278 10.1038/s41598-024-80610-z PMC11589107

[34] McKinnon C, Nandhabalan M, Murray SA, Plaha P (2021) Glioblastoma: clinical presentation, diagnosis, and management. BMJ 374:n1560 34261630 10.1136/bmj.n1560

[35] Melnick K, Miller P, Carmichael E, Wang Y, Tran D, Kresak JL, Ghiaseddin A, Rahman M (2022) Histologic findings at the time of repeat resection predicts survival in patients with glioblastoma. World Neurosurg 168:e451–e459 36206964 10.1016/j.wneu.2022.09.128

[36] Mirzayeva LS, Uçar M, Budak SN, Kaymaz AM, Yaylı N (2025) Pushing the boundaries of neurosurgical oncology: evaluating the superiority of supratotal resection over gross total resection in intraoperative MRI-guided glioma surgery. Neurosurg Rev 48(1):200 39909935 10.1007/s10143-025-03301-x PMC11799004

[37] Neska-Matuszewska M, Bladowska J, Sąsiadek M, Zimny A (2018) Differentiation of glioblastoma multiforme, metastases and primary central nervous system lymphomas using multiparametric perfusion and diffusion MR imaging of a tumor core and a peritumoral zone-Searching for a practical approach. PLoS One 13(1):e0191341 29342201 10.1371/journal.pone.0191341 PMC5771619

[38] Niyazi M, Andratschke N, Bendszus M et al (2023) ESTRO-EANO guideline on target delineation and radiotherapy details for glioblastoma. Radiother Oncol 184:109663 37059335 10.1016/j.radonc.2023.109663

[39] Otani Y, Higaki F, Fujii K et al (2024) Comparative analysis of intraoperative MRI and early postoperative MRI findings in glioma surgery patients. J Neurosurg 1-9. 10.3171/2024.7.JNS24784 10.3171/2024.7.JNS24784 39729586

[40] Otto KM, Ehricke H-H, Kumar V, Klose U (2013) Angular smoothing and radial regularization of ODF fields: application on deterministic crossing fiber tractography. Phys Med 29(1):17–32 22051017 10.1016/j.ejmp.2011.10.002

[41] Ozawa M, Brennan PM, Zienius K, Kurian KM, Hollingworth W, Weller D, Grant R, Hamilton W, Ben-Shlomo Y (2019) The usefulness of symptoms alone or combined for general practitioners in considering the diagnosis of a brain tumour: a case-control study using the clinical practice research database (CPRD) (2000–2014). BMJ Open 9(8):e029686 31471440 10.1136/bmjopen-2019-029686 PMC6720478

[42] Qiu Y, Li Y, Jiang C et al (2024) Toxicity and efficacy of different target volume delineations of radiation therapy based on the updated radiation therapy oncology group/national research group and European organization for research and treatment of cancer guidelines in patients with grade 3–4 glioma: a randomized controlled clinical trial. Int J Radiat Oncol Biol Phys. 10.1016/j.ijrobp.2024.11.094 39615657 10.1016/j.ijrobp.2024.11.094

[43] Rivera CA, Bhatia S, Morell AA et al (2024) Metabolic signatures derived from whole-brain MR-spectroscopy identify early tumor progression in high-grade gliomas using machine learning. J Neurooncol 170(3):579–589 39180640 10.1007/s11060-024-04812-1 PMC11614968

[44] Rykkje AM, Carlsen JF, Larsen VA, Skjøth-Rasmussen J, Christensen IJ, Nielsen MB, Poulsen HS, Urup TH, Hansen AE (2024) Prognostic relevance of radiological findings on early postoperative MRI for 187 consecutive glioblastoma patients receiving standard therapy. Sci Rep 14(1):10985 38744979 10.1038/s41598-024-61925-3 PMC11094076

[45] Sakata A, Okada T, Yamamoto A et al (2015) Grading glial tumors with amide proton transfer MR imaging: different analytical approaches. J Neurooncol 122(2):339–348 25559689 10.1007/s11060-014-1715-8

[46] Schön S, Cabello J, Liesche-Starnecker F et al (2020) Imaging glioma biology: spatial comparison of amino acid PET, amide proton transfer, and perfusion-weighted MRI in newly diagnosed gliomas. Eur J Nucl Med Mol Imaging 47(6):1468–1475 31953672 10.1007/s00259-019-04677-x PMC7188730

[47] Senger KPS, Kesavadas C, Thomas B, Singh A, Multani GS, An D, Label M, Suchandrima B, Shin D (2023) Experimenting with ASL-based arterialized cerebral blood volume as a novel imaging biomarker in grading glial neoplasms. Neuroradiol J 36(6):728–735 37548164 10.1177/19714009231193163 PMC10649543

[48] Sourbron SP, Buckley DL (2013) Classic models for dynamic contrast-enhanced MRI. NMR Biomed 26(8):1004–1027 23674304 10.1002/nbm.2940

[49] Sourbron S, Ingrisch M, Siefert A, Reiser M, Herrmann K (2009) Quantification of cerebral blood flow, cerebral blood volume, and blood-brain-barrier leakage with DCE-MRI. Magn Reson Med 62(1):205–217 19449435 10.1002/mrm.22005

[50] Suh CH, Kim HS, Jung SC, Choi CG, Kim SJ (2018) 2-Hydroxyglutarate MR spectroscopy for prediction of isocitrate dehydrogenase mutant glioma: a systemic review and meta-analysis using individual patient data. Neuro Oncol 20(12):1573–1583 30020513 10.1093/neuonc/noy113 PMC6231199

[51] Suh CH, Kim HS, Jung SC, Park JE, Choi CG, Kim SJ (2019) MRI as a diagnostic biomarker for differentiating primary central nervous system lymphoma from glioblastoma: a systematic review and meta-analysis. J Magn Reson Imaging 50(2):560–572 30637843 10.1002/jmri.26602

[52] Tang PLY, Romero AM, Nout RA, van Rij C, Slagter C, Swaak-Kragten AT, Smits M, Warnert EAH (2024) Amide proton transfer-weighted CEST MRI for radiotherapy target delineation of glioblastoma: a prospective pilot study. Eur Radiol Exp 8(1):123 39477835 10.1186/s41747-024-00523-4 PMC11525355

[53] Vallatos A, Al-Mubarak HFI, Birch JL, Galllagher L, Mullin JM, Gilmour L, Holmes WM, Chalmers AJ (2019) Quantitative histopathologic assessment of perfusion MRI as a marker of glioblastoma cell infiltration in and beyond the peritumoral edema region. J Magn Reson Imaging 50(2):529–540 30569620 10.1002/jmri.26580

[54] Villanueva-Meyer JE, Wood MD, Choi BS, Mabray MC, Butowski NA, Tihan T, Cha S (2018) MRI features and IDH mutational status of grade II diffuse gliomas: impact on diagnosis and prognosis. AJR Am J Roentgenol 210(3):621–628 29261348 10.2214/AJR.17.18457 PMC5823758

[55] Wakabayashi M, Ogiwara T, Ito K, Sato A, Hanaoka Y, Kobayashi K, Shimizu Y, Hongo K (2025) Glioblastoma mimicking autoimmune meningitis in an adult: a complex diagnostic challenge. Surg Neurol Int 16:61 40041069 10.25259/SNI_876_2024 PMC11878707

[56] Wen PY, van den Bent M, Youssef G et al (2023) RANO 2.0: update to the response assessment in neuro-oncology criteria for high- and low-grade gliomas in adults. J Clin Oncol 41(33):5187–5199 37774317 10.1200/JCO.23.01059 PMC10860967

[57] Woodall RT, Sahoo P, Cui Y et al (2021) Repeatability of tumor perfusion kinetics from dynamic contrast-enhanced MRI in glioblastoma. Neurooncol Adv 3(1):vdab174 34988454 10.1093/noajnl/vdab174 PMC8715899

[58] Zaccagna F, Riemer F, Priest AN et al (2019) Non-invasive assessment of glioma microstructure using VERDICT MRI: correlation with histology. Eur Radiol 29(10):5559–5566 30888488 10.1007/s00330-019-6011-8 PMC6719328

